# Executive dysfunction as a transdiagnostic mechanism of psychopathology: a neurocognitive framework for diagnosis and intervention

**DOI:** 10.3389/fnhum.2026.1778271

**Published:** 2026-04-02

**Authors:** Sandra Carvalho

**Affiliations:** Psychological Neuroscience Laboratory, Psychology Research Centre (CIPsi), Department of Basic Psychology, School of Psychology, University of Minho, Braga, Portugal

**Keywords:** cognitive control, executive dysfunction, hot and cold executive functions, neurocognitive mechanisms, personalized intervention, transdiagnostic psychopathology

## Abstract

Psychiatric disorders are typically classified using categorical diagnostic systems based on symptom clusters. However, these frameworks often fail to capture the cognitive and neurobiological mechanisms that transcend diagnostic boundaries and influence everyday functioning. Executive dysfunction, encompassing impairments in inhibitory control, working memory, cognitive flexibility, planning, and emotion regulation, was historically linked to focal damage in prefrontal brain regions. Converging evidence from neuropsychology, cognitive neuroscience, genetics, and clinical research indicates that executive dysfunction is prevalent across multiple psychiatric conditions and closely linked to functional impairment. This theory-driven article proposes that executive dysfunction represents a transdiagnostic dimension of psychopathology reflecting a shared neurocognitive vulnerability. Disruptions in both “cool” executive processes (e.g., cognitive control and working memory) and “hot” executive processes (e.g., emotion regulation and motivational control) may constitute a common pathway through which diverse psychiatric disorders impair adaptive functioning. Executive dysfunction is further conceptualized as an intermediate phenotype linking genetic liability, distributed neural circuit disruption, and everyday behavioral regulation. This perspective supports a shift toward transdiagnostic, mechanism-based mental health interventions that prioritize executive functioning as a central target for improving real-world functioning and long-term recovery.

## 1 Introduction

Mental health conditions have traditionally been classified using categorical diagnostic systems, most notably the Diagnostic and Statistical Manual of Mental Disorders (DSM) of the American Psychiatric Association and the International Classification of Diseases (ICD) of the World Health Organization. These frameworks define disorders based on clusters of observable symptoms, offering important advantages for clinical communication, service organization, and diagnostic standardization. However, such classification systems do not fully align with the underlying cognitive and neurobiological mechanisms that often cut across diagnostic boundaries.

In everyday clinical practice, categorical diagnostic systems often struggle with high comorbidity, substantial heterogeneity within diagnoses, and persistent difficulties in real-world functioning. These limitations have stimulated growing interest in transdiagnostic and dimensional perspectives, such as the Research Domain Criteria (RDoC) framework, which emphasizes core neurocognitive systems rather than focusing solely on symptom clusters. At the same time, the development of treatments has largely remained diagnosis-driven, raising questions about whether current intervention models adequately target the mechanisms underlying functional impairment.

Consistent with this concern, many existing treatments continue to produce only modest effects. An extensive umbrella review by Leichsenring et al. (2022) analyzed more than 100 meta-analyses including over 650,000 participants and reported small effect sizes for both psychotherapies (standardized mean difference of approximately 0.34) and pharmacotherapies (approximately 0.36) compared with placebo or treatment as usual. Importantly, these effects primarily reflect symptom reduction rather than sustained improvements in everyday functioning. Moreover, interpretation of these findings is complicated by methodological limitations, including weak control conditions, variable risk of bias, and publication bias. Together, these results suggest that current treatment models may be approaching their limits and underscore the need for mechanism-focused, functionally oriented approaches that address the processes constraining patients’ daily lives.

Executive functions (EF) refer to a set of higher-order cognitive processes that support the regulation of thought and behavior in the service of goal-directed action. Core components include inhibitory control, working memory, and cognitive flexibility, which enable individuals to organize behavior, adapt to environmental demands, and function effectively in everyday contexts (Diamond, 2013; Friedman and Miyake, 2017). More broadly, executive functioning reflects the capacity to deploy these processes in accordance with internal goals and contextual demands (Doebel, 2020), whereas the term “executive control” is often used to refer to the neural mechanisms and distributed control systems, particularly within prefrontal networks, that coordinate these processes across the brain (Miller and Cohen, 2001; Niendam et al., 2012). The organization of executive functioning has been described in several theoretical models, including the Unity/Diversity framework, which conceptualizes inhibition, shifting, and updating as distinct but interrelated components (Miyake et al., 2000; Friedman and Miyake, 2017), and hierarchical models emphasizing higher-order regulatory processes such as energization, task setting, and monitoring in the coordination of goal-directed behavior (Stuss and Alexander, 2007; Stuss, 2011).

Recent findings from genetics and neuroimaging increasingly challenge strictly categorical models of psychopathology. Large-scale cross-disorder genomic studies reveal substantial genetic overlap across psychiatric conditions previously considered distinct. Analyses from the Psychiatric Genomics Consortium indicate that shared genetic liability across major disorders is better captured by a small number of latent dimensions, accompanied by a higher-order general factor of psychopathology (Grotzinger et al., 2026). These genetic dimensions appear to show stronger associations with cognitive and functional traits than with traditional symptom-based diagnostic categories. Evidence from neuroimaging studies points in a similar direction. Meta-analytic investigations of cognitive control across psychiatric disorders identify overlapping disruptions in large-scale brain networks, particularly within frontoparietal and cingulo-opercular systems involved in executive control (McTeague et al., 2017). Within dimensional frameworks such as the RDoC, these findings suggest that shared biological vulnerability may partly manifest through alterations in core neurocognitive systems rather than through mechanisms unique to specific disorders (Insel et al., 2010). Despite growing evidence for shared biological vulnerability, the cognitive mechanisms through which these vulnerabilities translate into persistent functional impairment remain incompletely understood. Neural executive control systems therefore represent a strong candidate mechanism linking biological risk to real-world functional outcomes.

However, understanding the role of executive control systems in psychopathology also requires clarifying the internal organization of executive functions themselves. Executive function models have traditionally been conceptualized as reflecting stable, domain-general cognitive constructs. However, emerging evidence suggests that observed executive function structures may partly reflect characteristics of the tasks used to measure them, including methodological similarities and task-specific variance (Sambol et al., 2023). For example, Sambol et al. (2023) report that traditional unity/diversity models may not fully capture the empirical structure of executive functioning, instead supporting an alternative organization characterized by expanded working memory, integrated cognitive flexibility, and substantial task-specific variance. These findings highlight the need for frameworks that more closely capture how executive capacities operate in real-world contexts and how they relate to functional outcomes. Across theoretical perspectives, executive functions are widely regarded as essential for adaptive, goal-directed behavior (Diamond, 2013; Friedman and Miyake, 2017; Miller and Cohen, 2001). Consistent with this view, impairments in executive functioning are reported across a wide range of psychiatric conditions, including ADHD, schizophrenia, autism spectrum disorder, mood disorders, and substance use disorders (Insel et al., 2010; Snyder et al., 2015). Such deficits are strongly associated with long-term academic, occupational, and social outcomes and often predict functional impairment more reliably than symptom severity alone (Millan et al., 2012; Romer and Pizzagalli, 2021).

At the neural level, executive processes are supported by distributed prefrontal and fronto-subcortical circuits. The dorsolateral prefrontal cortex (dlPFC) plays a central role in working memory, cognitive control, and the maintenance of goal representations that guide behavior (Miller and Cohen, 2001), while other regions such as the anterior cingulate cortex and ventrolateral prefrontal cortex contribute to conflict monitoring, error detection, response inhibition, and cognitive flexibility (Niendam et al., 2012; Stuss, 2011). Evidence from lesion studies and functional neuroimaging further indicates that executive functioning emerges from the coordinated activity of distributed neural systems, including the frontoparietal control network, the cingulo-opercular network, and subcortical structures such as the basal ganglia and cerebellum (Bostan et al., 2013; Duncan, 2010; Seeley et al., 2007). Taken together, these findings suggest that neural executive control systems may represent a key neurocognitive mechanism through which shared biological vulnerabilities translate into functional impairment across psychiatric disorders.

## 2 Hypothesis: executive dysfunction as a transdiagnostic core

Mental disorders differ widely in their clinical presentation and diagnostic criteria, yet converging evidence suggests that executive dysfunction represents a shared cognitive vulnerability across psychiatric conditions (Diamond, 2013; Snyder et al., 2015). Difficulties in sustaining goal-directed behavior, regulating attention and emotion, organizing actions over time, inhibiting impulsive responses, and flexibly adjusting behavior are repeatedly observed across diagnoses, indicating disruptions in executive functioning and in the executive control systems that support it, rather than disorder-specific deficits. These difficulties should not be interpreted solely as secondary consequences of disorder-specific symptoms. Instead, they may reflect a fundamental mechanism through which psychopathology disrupts adaptive functioning, contributing to disorganization, impulsivity, emotional dysregulation, and difficulties in social and occupational contexts (McTeague et al., 2016; Snyder et al., 2015).

Conceptualizing executive dysfunction as a “core” feature of psychopathology does not imply that it represents the sole causal mechanism underlying all mental disorders. Rather, it refers to a central regulatory process within the broader architecture of behavior and self-regulation. Executive functions coordinate attention, goal maintenance, action selection, emotion regulation, and adaptive decision-making across contexts. Because of this integrative role, disruptions in executive functioning can influence multiple cognitive, emotional, and behavioral processes simultaneously. In this sense, executive dysfunction may operate as a hub-like mechanism linking shared biological vulnerabilities, disruptions in distributed neural circuits, and impairments in real-world functioning across diagnostic categories. Consistent with this interpretation, executive functioning is among the most reliable predictors of real-world outcomes across psychiatric conditions, including academic and occupational functioning, treatment engagement, and illness course (Millan et al., 2012; Romer and Pizzagalli, 2021; Snyder et al., 2015).

Within a transdiagnostic framework, a process may be considered “core” when several criteria are met. It should be observable across multiple diagnostic categories, demonstrate a consistent relationship with real-world functioning, and reflect alterations in shared neural systems rather than mechanisms unique to specific diagnoses. In addition, the process should be modifiable through intervention, allowing it to serve as a meaningful target for treatment. Executive dysfunction satisfies many of these criteria: it is consistently documented across psychiatric conditions, closely associated with everyday functioning, linked to distributed control networks in the brain, and increasingly targeted in cognitive and behavioral interventions. Evidence from meta-analyses and population-based studies further indicates that executive impairments are prevalent and clinically significant across disorders including ADHD, major depressive disorder, bipolar disorder, obsessive-compulsive disorder, post-traumatic stress disorder, borderline personality disorder, schizophrenia, and substance use disorders (Kessler et al., 2007; Martel et al., 2016). Across these conditions, deficits in executive functioning are consistently associated with reduced academic and occupational performance, impaired interpersonal functioning, diminished self-regulatory capacity, and poorer long-term outcomes (Millan et al., 2012; Snyder et al., 2015).

Dimensional research frameworks, particularly the Research Domain Criteria (RDoC), highlight the importance of executive functioning as a central component of cognitive control and self-regulation within broader functional systems. These perspectives encourage a shift away from strictly categorical models of psychopathology toward an understanding of shared neurocognitive processes that cut across diagnostic boundaries (Insel et al., 2010; McTeague et al., 2016). Within this framework, executive dysfunction can be conceptualized not merely as a consequence of psychiatric illness but as a transdiagnostic cognitive phenotype contributing to vulnerability, chronicity, and functional outcomes.

In the present article, executive dysfunction is therefore proposed as a central cognitive dimension spanning multiple mental health conditions, including ADHD, schizophrenia, mood disorders, autism spectrum disorder, and substance use disorders. Both “cool” executive processes (e.g., working memory, inhibitory control, and planning) and “hot” executive processes (e.g., emotion regulation and motivational control) may contribute to functional impairment when these systems are compromised. This perspective is also consistent with clinical observations: across diverse diagnoses, individuals frequently experience similar difficulties in planning, decision-making, task initiation, sustaining effort, and regulating behavior in everyday contexts. Taken together, empirical evidence and clinical observations suggest that executive dysfunction represents not merely a secondary feature of psychopathology but a key mechanism shaping both symptom expression and recovery trajectories. Viewing executive functioning as a central diagnostic and therapeutic target may therefore support more personalized and ecologically valid models of psychiatric care.

Genetic and environmental risk factors are proposed to converge on shared neurobiological systems, particularly prefrontal–subcortical control networks involved in executive functioning. Disruptions within these systems may produce transdiagnostic executive dysfunction, measurable through behavioral, neurophysiological, and neuroimaging indicators. Variations in executive functioning are hypothesized to influence symptom expression and real-world functional outcomes across diagnostic categories. The model is organized around three propositions: (H1) shared etiological risk converges on executive systems, leading to transdiagnostic executive dysfunction; (H2) executive functioning predicts functional outcomes beyond symptom severity; and (H3) executive dysfunction reflects disruption in distributed prefrontal–subcortical control networks. The framework is illustrated across several psychiatric conditions, including mood, obsessive-compulsive and related, attention-deficit/hyperactivity, psychotic, personality, and addictive disorders.

### 2.1 Core propositions of the hypothesis

First, executive dysfunction is a common feature observed across multiple psychiatric conditions, transcending traditional diagnostic boundaries. Despite significant differences in how symptoms may present, disorders such as ADHD, mood and psychotic disorders, obsessive-compulsive disorder, autism spectrum disorder, personality disorders, and substance use disorders frequently show impairments in key executive functions. These impairments include difficulties in cognitive control, planning, impulse regulation, working memory, and flexible adaptation. These challenges often result in similar cognitive and behavioral patterns across different diagnoses. This suggests that executive dysfunction should be viewed as a shared vulnerability rather than a deficit specific to any one disorder.

Second, executive functioning is a significant and clinically important predictor of real-world functioning, often providing greater insight than the severity of symptoms. Deficits in executive functioning are consistently associated with difficulties in academic and occupational performance, social integration, independent living, and overall autonomy. Furthermore, executive dysfunction has been linked to factors such as treatment engagement, adherence, the risk of relapse, and long-term psychosocial outcomes. This highlights its importance as a key focus for both assessment and intervention (Romer and Pizzagalli, 2021). Thus, executive functioning serves as a critical factor in everyday functioning, addressing aspects of mental health issues that symptom-based measures typically do not capture.

Third, recent neurobiological research suggests that shared neural circuits contribute to executive dysfunction across various psychiatric conditions. Disruptions in areas such as the dorsolateral and ventrolateral prefrontal regions, the anterior cingulate cortex, and fronto-subcortical control loops have consistently been linked to executive impairments across various diagnostic categories. These findings suggest that changes in distributed control networks serve as a common mechanism for transdiagnostic executive dysfunction. This implies that difficulties in goal-directed behavior and self-regulation result from similar disruptions in neural systems, rather than being limited to specific disorders (Miller and Cohen, 2001; Niendam et al., 2012; Stuss, 2011).

An important implication of this framework is that interventions targeting executive functioning and the neural executive control systems that support it may offer benefits across various conditions. Approaches that combine executive function training with noninvasive brain stimulation targeting prefrontal control networks represent a promising strategy to enhance functional outcomes in psychiatric disorders. By strengthening core executive processes, such as working memory, inhibitory control, cognitive flexibility, and emotion regulation, these interventions may increase self-regulatory capacity, improve engagement with psychotherapy and medication treatments, and reduce the risk of relapse. This hypothesis does not suggest that these interventions are disorder-specific; rather, it proposes that improvements in executive functioning may act as a common mechanism through which symptoms, behavior, and adaptive functioning can be positively influenced across conditions. Conceptualizing executive dysfunction as a transdiagnostic cognitive mechanism therefore provides a coherent framework for integrating clinical, cognitive, and neuroscientific evidence. Within this perspective, executive dysfunction links neural circuitry, behavior, and real-world functioning, highlighting executive capacity and adaptive control as central targets for assessment and intervention. Emphasizing these processes extends beyond symptom-based models and offers a framework for understanding vulnerability, persistence, and recovery across psychiatric conditions (Insel et al., 2010).

## 3 Supporting evidence: shared executive function deficits across mental health conditions

Evidence supporting the transdiagnostic role of executive dysfunction can be organized across several complementary dimensions, including its presence across multiple psychiatric conditions, its influence on the onset and course of psychopathology, its underlying neurocognitive and neural mechanisms, and the clinical implications of targeting executive processes in intervention. Findings from neuropsychological, longitudinal, and neuroscientific research converge in suggesting that executive dysfunction represents a shared mechanism contributing to functional impairment across psychiatric disorders.

Across neuropsychological, and neuroscientific studies, executive dysfunction consistently emerges across a wide range of psychiatric disorders. Although the clinical presentation of these conditions varies considerably, a recurring pattern of impairments is observed in core executive processes such as inhibitory control, cognitive flexibility (set-shifting), planning, working memory, and the regulation of emotion and behavior. These impairments are not only prevalent but also clinically consequential, as executive deficits have been repeatedly associated with functional outcomes, treatment responsiveness, and the long-term course of psychiatric illness. Across conditions including ADHD, mood and anxiety disorders, obsessive-compulsive disorder, psychotic disorders, personality disorders, and substance use disorders, such deficits can limit the capacity to organize behavior over time, regulate emotions, respond adaptively to environmental demands, and sustain engagement in goal-directed activity (McTeague et al., 2016; Millan et al., 2012; Snyder et al., 2015).

Evidence further indicates that executive dysfunction is often a stronger predictor of real-world functioning than measures focused exclusively on symptom severity. Executive impairments have been closely linked to outcomes such as academic performance, occupational success, social functioning, and independent living (Barkley, 2012; Romer and Pizzagalli, 2021). This pattern suggests that executive dysfunction may operate as a proximal determinant of adaptive capacity, influencing how individuals manage everyday cognitive and emotional demands. In this sense, executive dysfunction can be conceptualized as a transdiagnostic neurocognitive constraint on adaptive functioning, contributing to functional difficulties across a wide range of psychiatric conditions.

Personality disorders provide substantial evidence for the transdiagnostic relevance of executive dysfunction. A systematic review by Garcia-Villamisar et al. (2017) identified consistent patterns of executive impairments across several personality disorders, including borderline, obsessive-compulsive, antisocial, narcissistic, and schizotypal personality disorders. Across these conditions, deficits were most consistently observed in decision-making, working memory, inhibitory control, and cognitive flexibility, core executive processes that support behavioral regulation and adaptive responding. Such impairments likely reflect disruptions in prefrontal executive control mechanisms and contribute to characteristic features of personality pathology, including behavioral dysregulation, impulsivity, cognitive rigidity, and interpersonal difficulties. The substantial overlap of executive deficits across different personality disorders therefore suggests that executive dysfunction represents a common neurocognitive vulnerability rather than a disorder-specific abnormality.

Empirical studies further support the relevance of executive impairment in personality pathology. In a cross-sectional investigation, Shankar et al. (2025) reported strong associations between executive dysfunction, reduced working memory capacity, and increased impulsivity in individuals with borderline personality disorder. Executive deficits were also related to the occurrence of deliberate self-harm behaviors. Specifically, perseverative errors on the Wisconsin Card Sorting Test and reduced working memory capacity were significant predictors of impulsivity severity. These findings illustrate how disruptions in executive functioning and its underlying control mechanisms can translate into maladaptive behavioral outcomes. Moreover, similar executive impairments observed across borderline personality disorder and substance use disorders further reinforce the notion that executive dysfunction extends across diagnostic boundaries.

Substance use disorders provide additional empirical support for this perspective. A comprehensive synthesis by Brockett et al. (2018) documented that chronic exposure to addictive substances is associated with impairments in several key executive functions, including reversal learning, delay discounting, and response inhibition. These deficits are particularly pronounced during acute withdrawal and craving periods and are linked to reduced regulatory capacity within the prefrontal cortex, increased behavioral impulsivity, and elevated relapse risk. At the neural level, these alterations involve disruptions in prefrontal and cortico-striatal circuits, including the orbitofrontal cortex, anterior cingulate cortex, and striatum. Similar prefrontal abnormalities are reported across multiple psychiatric conditions, suggesting that executive dysfunction may represent a shared neurobiological pathway linking substance use disorders with broader psychiatric vulnerability through impaired cognitive control and behavioral regulation.

Longitudinal research further clarifies the functional role of executive dysfunction in addictive behaviors. In a prospective study of young adults, Kräplin et al. (2022) found that baseline executive functioning did not strongly differentiate individuals with substance-related or behavioral addictions from controls. However, lower executive functioning predicted the progression of maladaptive use patterns over time, including greater increases in consumption and slower reductions in frequency of use. Executive functioning was more closely associated with the escalation and persistence of addictive behaviors, particularly with the loss of behavioral control, than with the categorical presence of DSM-5 addiction diagnoses. These findings suggest that executive dysfunction may influence behavioral trajectories and regulatory capacity rather than serving as a categorical vulnerability marker for addiction.

Evidence from mood and anxiety disorders also highlights the role of executive dysfunction, although its direction of influence may vary depending on the clinical context. Using cross-lagged panel network analysis in a large community sample of older adults, Zainal and Newman (2023) found that elevated levels of anxious and depressed mood predicted subsequent declines in executive functioning, including working memory, attention, and processing speed. In contrast, executive functioning showed limited predictive influence on later mood symptoms. These findings are consistent with scar models of psychopathology, which propose that prolonged emotional distress may progressively compromise neural executive control systems through mechanisms such as chronic stress exposure, inflammatory processes, and vulnerability in fronto-cortical networks. Within this framework, executive dysfunction appears as a key outcome linking emotional symptoms to broader cognitive and functional decline.

Experimental studies in non-clinical populations provide additional insight into which components of executive functioning are most closely involved in emotion regulation. A two-study investigation by Rodas et al. (2024) examined the contributions of working memory, inhibitory control, and cognitive flexibility to emotional downregulation using performance-based measures. Across both studies, cognitive flexibility—specifically the ability to shift between mental strategies—emerged as the only consistent predictor of successful emotional downregulation. In contrast, working memory and inhibitory control did not show robust associations with regulation outcomes. These findings suggest that effective emotion regulation may depend particularly on the capacity to flexibly adapt cognitive strategies in response to changing emotional contexts. This pattern aligns with transdiagnostic frameworks proposing that impairments in cognitive flexibility represent a key mechanism linking executive dysfunction to emotional dysregulation and broader functional impairment.

In sum, these findings indicate that impairments in executive functioning are not confined to any single diagnostic category but instead reflect shared cognitive mechanisms that cut across multiple psychiatric conditions. Difficulties in regulating behavior, emotions, and goal-directed activity appear to represent a central pathway through which diverse forms of psychopathology disrupt everyday functioning. This convergence of evidence supports the conceptualization of executive dysfunction as a fundamental transdiagnostic dimension of mental disorders.

### 3.1 Working memory

Working memory refers to the capacity to maintain and manipulate goal-relevant information over short periods in order to guide behavior and support the coordination of complex cognitive operations. This system plays a central role in planning, decision-making, and the regulation of goal-directed behavior, as it allows individuals to keep task goals active while integrating incoming information. Across psychiatric conditions, impairments in working memory represent one of the most consistently documented cognitive deficits and are strongly associated with functional outcomes in everyday life.

Disruptions in working memory capacity can therefore contribute directly to a range of cognitive and behavioral difficulties. When individuals have limited ability to maintain and update goal-relevant information, planning efficiency is reduced and the pursuit of goals becomes more effortful and unstable. In clinical contexts such as depression, constraints on working memory resources may hinder the ability to keep task goals active while simultaneously processing emotional or contextual information. This imbalance can lead to slowed decision-making, indecisiveness, and difficulties initiating or completing tasks. As a result, working memory deficits are closely linked to impairments in everyday functioning, including occupational performance, problem-solving capacity, and persistence in goal-directed activities (Barch and Ceaser, 2012; Bowie and Harvey, 2006). Emotional context can substantially interfere with working memory processes. During negative emotional states, working memory resources may become occupied by mood-congruent thoughts, reducing the capacity available to maintain task goals and organize behavior. This competition for cognitive resources can promote maladaptive patterns such as rumination and weaken executive functioning. Consequently, executive functioning may appear relatively intact under low cognitive or emotional demands but deteriorate when individuals must sustain goal-directed control while simultaneously regulating emotional responses (Joormann and D’Avanzato, 2010).

Evidence across diagnostic groups supports the transdiagnostic relevance of these mechanisms. In schizophrenia and related disorders, impairments in working memory and goal maintenance are among the strongest predictors of functional outcomes and are closely associated with difficulties in planning, problem-solving, and everyday task execution (Barch and Ceaser, 2012; Bowie and Harvey, 2006). Large-scale reviews similarly identify working memory deficits as one of the most consistent cognitive abnormalities across psychiatric conditions, contributing to disability independently of symptom severity (Millan et al., 2012). Comparable patterns are observed in ADHD, where working memory capacity strongly predicts organizational skills, task planning, and the ability to sustain goal-directed behavior beyond the influence of core symptoms (Kofler et al., 2018). Neuroimaging findings further indicate reduced functional connectivity during working memory tasks across fronto-parietal, fronto-striatal, cerebellar, and subcortical networks, with the degree of hypoconnectivity correlating with difficulties in sustained attention and inhibitory control (Tolonen et al., 2024).

Similar findings have also been reported in obsessive-compulsive disorder. A systematic review by Tubío Fungueiriño et al. (2020) showed that working memory impairments become particularly evident under conditions of high cognitive load or increased executive demands, suggesting that these deficits reflect limitations in cognitive capacity rather than generalized cognitive decline. Overall, this body of evidence indicates that working memory plays a central role in maintaining goal-directed behavior across psychiatric conditions. When working memory capacity is compromised, individuals experience greater difficulty sustaining attention to goals, integrating contextual information, and coordinating complex behavior over time.

### 3.2 Inhibitory control

Impaired response inhibition is among the most consistently observed markers of executive dysfunction across psychiatric conditions. Clinical and neuropsychological studies show recurrent deficits in suppressing dominant motor responses, filtering irrelevant information, and regulating maladaptive thought patterns. Such impairments have been documented in ADHD, mood disorders, OCD, personality disorders, psychotic disorders, and substance use disorders. The recurrence of this phenotype across diagnostic categories suggests disruption of shared prefrontal–striatal circuits responsible for behavioral suppression rather than disorder-specific pathophysiological mechanisms.

ADHD provides one of the clearest examples of the functional relevance of inhibitory control deficits. In Barkley’s influential model, impaired behavioral inhibition is conceptualized as the central mechanism underlying broader disturbances in executive self-regulation, including deficits in working memory, attentional persistence, and goal-directed behavior (Barkley, 1997). Meta-analytic evidence confirms robust impairments in inhibitory control and working memory in ADHD, and these deficits predict functional outcomes in academic, occupational, and social domains beyond the severity of core symptoms (Kofler et al., 2017; Willcutt et al., 2005). Similar patterns are observed in mood disorders, where inhibitory control deficits contribute to cognitive disturbances such as rumination, psychomotor slowing, and reduced processing efficiency. These impairments often persist even during remission phases, suggesting that they reflect a trait-like vulnerability rather than transient state effects (Snyder, 2013).

Failures of inhibitory control are also central to disorders characterized by maladaptive repetitive behavior. In obsessive-compulsive disorder, intrusive thoughts are difficult to suppress and habitual behavioral responses become difficult to disengage once initiated. A related mechanism appears in substance use disorders, where weakened prefrontal inhibitory control fails to counteract the salience of reward-related dopaminergic signals, increasing vulnerability to craving and relapse despite explicit intentions to abstain (Bickel et al., 2012; Goldstein and Volkow, 2011). Across these conditions, similar failures of behavioral inhibition manifest as compulsive actions, loss of control, or progressive functional decline.

At the neurobiological level, inhibitory control depends on a distributed fronto-subcortical network. The inferior frontal gyrus (IFG), particularly in the right hemisphere, plays a central role in both proactive and reactive inhibitory processes. Converging evidence from neuroimaging, lesion studies, and brain stimulation demonstrates that the IFG contributes to anticipating, suppressing, and modulating prepotent responses within a broader network involving the basal ganglia and other prefrontal regions. Causal evidence from non-invasive brain stimulation further supports this role. In our work, transcranial direct current stimulation targeting the IFG modulated proactive inhibition depending on electrode montage: unihemispheric stimulation over the right IFG shifted the speed–accuracy trade-off toward greater response caution, whereas bihemispheric stimulation abolished this effect, highlighting the importance of interhemispheric balance in inhibitory control mechanisms (Tubío Fungueiriño et al., 2020).

Developmental evidence also indicates that inhibitory control deficits may arise from early vulnerabilities in prefrontal systems. In adolescents with early-onset schizophrenia, Teigset et al. (2020) found that exposure to perinatal obstetric complications, particularly shorter gestational length, was associated with increased perseveration, reduced cognitive inhibition, and impaired problem-solving performance. These associations were present in patients but not in healthy controls exposed to similar perinatal conditions, suggesting heightened neurodevelopmental susceptibility of executive control systems in early psychosis. Lower Apgar scores were also associated with later executive dysfunction in both groups, indicating that early compromise of neonatal physiological integrity may constitute a general risk factor for later inhibitory control difficulties.

Taken together, evidence from behavioral, clinical, neurobiological, and neuromodulation research indicates that inhibitory control represents a central component of executive dysfunction across psychiatric disorders. Its strong association with functional decline, treatment response, and relapse highlights its relevance not only as a cognitive marker but also as a potential therapeutic target. Interventions aimed at strengthening inhibitory control, through cognitive remediation, neuromodulation techniques, or multimodal treatment approaches, may therefore offer benefits across diagnostic boundaries by engaging shared neural executive control circuitry.

### 3.3 Cognitive flexibility

Impairments in cognitive flexibility, often operationalized in neuropsychological tasks as set-shifting, reflect reduced capacity to adapt behavior and switch between rules, strategies, or mental representations, and represent a core transdiagnostic feature of executive dysfunction. Across various psychiatric conditions, deficits in set-shifting manifest as cognitive rigidity, perseveration, and difficulties in updating rules, strategies, or goals in response to new information. These impairments have been consistently observed in conditions such as OCD, mood disorders, schizophrenia-spectrum disorders, personality disorders, and substance use disorders. This suggests a common vulnerability in adaptive cognitive control, rather than anomalies that are specific to each disorder.

In individuals with OCD, research shows significant impairments in cognitive flexibility and set-shifting. These deficits are believed to contribute directly to behavioral rigidity, compulsive repetition, and challenges in disengaging from habitual action patterns (Abramovitch et al., 2013; Snyder et al., 2015). Similarly, people with mood disorders, particularly major depressive disorder and bipolar disorder, also show reduced cognitive flexibility. This is evident through impaired problem-solving abilities, diminished sensitivity to feedback, and difficulty adjusting behavior in response to emotional or contextual changes. Executive deficits in mood disorders are often observed even during periods of remission or when symptoms are not present, suggesting that these cognitive challenges may be more of a stable trait rather than purely linked to the current mood state.

A large-scale meta-analysis conducted by Cotrena et al. (2020) found that both types of bipolar disorder (type I and type II) are linked to significant impairments in various executive functions, such as cognitive flexibility and set-shifting. These deficits persist regardless of acute mood symptoms, indicating that executive dysfunction is a stable aspect of the neurocognitive profile for individuals with bipolar disorder, rather than a temporary condition associated with mood episodes. In the case of borderline personality disorder, meta-analytic evidence shows impairments in several executive functions, including cognitive flexibility and planning. In a comprehensive meta-analysis, Ruocco (2005) found that individuals with borderline personality disorder performed significantly worse than healthy controls across tasks involving attention, cognitive flexibility, learning and memory, planning, and processing speed. While the effect sizes for cognitive flexibility were smaller compared to planning, the consistent pattern of deficits suggests that compromised executive functioning is likely linked to prefrontal systems. These impairments may lead to difficulties in revising goals, adapting behavior to changing circumstances, and regulating behavior in complex interpersonal situations.

This collection of research suggests that difficulties with set-shifting reflect a broader limitation in executive functioning, impacting individual behavior across emotional, cognitive, and social domains. This interpretation is bolstered by evidence from transdiagnostic studies. Specifically, comparative analyses of substance use disorders and behavioral addictions consistently highlight deficits in key components of executive functioning. These deficits manifest as impulsive actions (indicating poor response inhibition) and impulsive choices (favoring immediate gratification) (Grant and Chamberlain, 2014). Such deficits are observed in both substance-related and non-substance-related addictive behaviors, implying shared disruptions in the top-down regulation of habitual and reward-driven actions. Although much of the literature focuses primarily on inhibitory control and decision-making—rather than on set-shifting in a narrow sense—the consistent presence of executive dysfunction across various compulsive and addictive conditions supports a broader understanding of executive vulnerability. From this perspective, cognitive rigidity, perseveration, and maladaptive repetition may stem from common limitations in executive systems that go beyond traditional diagnostic categories.

Cognitive flexibility and set-shifting are supported by a distributed neural network involving the dorsolateral prefrontal cortex, anterior cingulate cortex, and basal ganglia. Disruptions within this circuitry have been consistently associated with conditions characterized by behavioral rigidity and compulsivity. Converging causal evidence further supports the functional relevance of this network. In our neuromodulation study, transcranial direct current stimulation (tDCS) applied to prefrontal and motor regions modulated set-shifting performance in a task-specific manner (Coelho et al., 2025). Changes in cortical excitability influenced the speed with which individuals switched between cognitive and motor sets, while error rates and shift costs remained largely unaffected. These findings suggest that set-shifting performance primarily reflects the efficiency and coordination of executive control networks rather than simple accuracy measures (Feeser et al., 2014). Moreover, the observation that stimulation of both prefrontal and motor regions influenced set-shifting performance indicates that cognitive flexibility is not strictly localized to discrete cortical sites. Instead, it appears to emerge from the coordinated activity of distributed task-relevant networks. This interpretation is consistent with converging evidence from neuroimaging, lesion, and electrophysiological studies demonstrating that successful set-shifting depends on dynamic interactions between cortical and subcortical systems, particularly basal ganglia circuits involved in updating and switching task representations.

There is increasing evidence that impairments in cognitive flexibility are influenced by emotional context, especially in disorders characterized by compulsivity and emotional dysregulation. For instance, in obsessive-compulsive disorder, Tubío Fungueiriño et al. (2020) demonstrated that deficits in set-shifting and inhibitory control were more pronounced when tasks involved negative emotional valence or aversive stimuli. These findings highlight the close relationship between executive functioning and emotional processing, suggesting that rigidity in cognitive flexibility is more about the influence of motivational and emotional states rather than simply a “cold” executive limitation. Similar interactions are likely to occur across conditions marked by rigidity, compulsivity, and difficulties in emotion regulation.

From a transdiagnostic perspective, deficits in cognitive flexibility serve as a key mechanism by which psychiatric disorders limit adaptive behavior. Difficulty in moving away from outdated rules, strategies, or emotional states hinders learning, promotes perseveration, and may restrict the effectiveness of therapeutic interventions. In this context, cognitive flexibility, especially set-shifting, represents a fundamental executive function that connects various clinical presentations through common neurocognitive and neurobiological pathways.

### 3.4 Planning and goal management

Planning and goal management refer to higher-order executive processes that allow individuals to organize sequences of actions, maintain long-term objectives, and monitor progress toward desired outcomes. These processes enable the translation of intentions into structured behavior and support the maintenance of goal-directed activity despite distractions or competing demands. They rely on sustained engagement of prefrontal control systems that coordinate behavior across time, allowing individuals to integrate contextual information, anticipate future consequences, and adjust actions in pursuit of abstract goals. Disruptions in these processes contribute substantially to functional impairment across psychiatric conditions. In mood disorders, for example, individuals with major depressive disorder frequently experience difficulties organizing actions over time and maintaining engagement with long-term goals. Such impairments can persist beyond acute mood episodes and interfere with the ability to translate intentions into effective behavior, adapt to changing circumstances, and sustain effort toward meaningful objectives. From a control systems perspective, these patterns reflect weakened engagement of prefrontal mechanisms responsible for maintaining goal representations and coordinating behavior over extended time scales. When executive functioning is compromised, behavior tends to become more reactive and less flexibly directed toward long-term goals, increasing vulnerability to behavioral inertia and reducing the capacity to initiate or sustain adaptive actions, including those required for therapeutic change.

Hierarchical models of prefrontal cortex organization provide a useful framework for understanding these difficulties. According to these models, the prefrontal cortex maintains abstract goals and task rules that guide processing across distributed neural systems (Miller and Cohen, 2001). Rostro–caudal accounts further propose that progressively anterior regions of the prefrontal cortex support increasingly abstract levels of control, coordinating behavior across longer time horizons and more complex contextual demands (Badre and D'Esposito, 2009). Disruptions within these hierarchical control systems therefore impair the capacity to structure behavior around long-term objectives.

Such impairments also influence behavioral regulation over time. When executive functioning is weakened, individuals may rely more heavily on habitual or stimulus-driven responses rather than flexible, goal-directed strategies. This shift from goal-directed control to habitual responding has been implicated in the persistence and relapse of maladaptive behaviors in conditions such as addiction and compulsive disorders (Everitt and Robbins, 2016). Across psychiatric disorders, including mood disorders, schizophrenia, ADHD, OCD, and substance use disorders, difficulties in planning and goal management therefore reflect shared constraints in executive functioning that extend beyond specific diagnostic categories. These deficits contribute directly to functional impairment, interact with emotional and motivational processes, and highlight planning and goal management as important targets for assessment and intervention within transdiagnostic models of executive dysfunction.

### 3.5 Emotion regulation and other forms of “hot” executive function

Emotion regulation is widely conceptualized as a central component of hot executive functioning, referring to the regulation of behavior when cognition interacts with emotionally or motivationally salient information (Zelazo and Carlson, 2012). In addition to emotion regulation, hot executive functions also encompass processes involved in emotional decision-making, reward evaluation, and socio-affective processing that guide behavior in motivationally relevant contexts. In such contexts, executive systems must maintain goal-directed control while interacting with affective and motivational processes that can bias attention, disrupt goal maintenance, or override planned behavior. Contemporary models therefore conceptualize hot executive functions as regulatory processes that guide behavior when emotional or reward-related signals compete with long-term goals, relying on interactions between prefrontal control systems and valuation-related circuits including the orbitofrontal cortex, ventromedial prefrontal cortex, and ventral striatum (Bechara, 2005; Bechara and Damasio, 2005; Rolls, 2019; Lin et al., 2025).

Within this framework, emotion regulation represents a central manifestation of hot executive functioning, reflecting the ability to maintain and implement regulatory goals despite heightened emotional demands (Ochsner and Gross, 2005; Zelazo and Carlson, 2012). Evidence from dimensional and highly comorbid samples indicates that emotion dysregulation is widely distributed across psychiatric conditions. For example, studies using the Difficulties in Emotion Regulation Scale (DERS) show that impairments in emotional regulation occur across multiple diagnostic groups, often differing in severity rather than in qualitatively distinct profiles (Aldao et al., 2010). Disorders characterized by impulsivity and reduced self-regulatory capacity, such as ADHD, frequently present pronounced emotion regulation difficulties (Barkley, 2015; Shaw et al., 2014), suggesting that emotional dysregulation reflects disruptions in executive–affective coordination rather than disorder-specific deficits.

At the neural level, emotion regulation depends on coordinated activity across prefrontal, limbic, and autonomic systems. The dlPFC supports the maintenance of regulatory goals and the implementation of cognitive strategies such as reappraisal, whereas ventromedial and medial prefrontal regions contribute to affective valuation and contextual integration (Etkin et al., 2011; Miller and Cohen, 2001). Neuroimaging studies further indicate that emotionally salient stimuli recruit distributed networks linking prefrontal regions with limbic and motivational systems (Gonçalves et al., 2015). Emotional dysregulation can therefore be conceptualized as a failure of executive systems to modulate limbic and reward-related reactivity, resulting in heightened emotional responses, impulsive behavior, and maladaptive coping.

Hot executive functions extend beyond emotion regulation to include processes related to reward sensitivity and emotional decision-making. Emotional decision-making refers to the selection of actions under conditions in which affective signals influence the evaluation of potential outcomes. This form of decision-making relies on interactions between executive control systems and neural circuits involved in reward valuation, particularly the orbitofrontal cortex, ventromedial prefrontal cortex, and ventral striatum (Bechara, 2005; Rolls, 2019). Converging evidence indicates that the ventromedial prefrontal cortex functions as a key hub linking value representation, emotional regulation, and social–affective processing through its interactions with limbic and striatal systems (Hiser and Koenigs, 2018). Alterations in these mechanisms have been documented across several psychiatric conditions. For instance, individuals with addiction frequently show impairments in reward evaluation and delay discounting, reflecting a shift toward immediate reward seeking at the expense of long-term goal-directed behavior (Everitt and Robbins, 2016).

Consistent with dimensional models of psychopathology, recent evidence suggests that reward dysfunction is associated not only with specific psychiatric disorders but also with general psychopathology. Using a large transdiagnostic sample of children and adults, Saxena et al. (2024) showed that multiple reward-processing measures—including delay discounting, reward responsiveness, and effort-based decision-making—were significantly associated with both the general psychopathology factor (P-factor) and specific symptom domains. Within the RDoC framework, such processes fall within the Positive Valence Systems, which encompass constructs such as reward valuation, reward learning, and reward responsiveness that guide behavior in motivationally relevant contexts (Cuthbert, 2014).

Taken together, these findings indicate that hot executive functions encompass a broader set of processes than emotion regulation alone, integrating emotional control with reward-related and emotional decision-making mechanisms, as well as other socio-affective processes that guide behavior in motivationally salient contexts.

## 4 Discussion

A growing body of evidence from neuropsychology, clinical psychiatry, and cognitive neuroscience indicates that executive dysfunction represents a significant dimension of psychopathology rather than a minor or disorder-specific feature. Across many psychiatric conditions, impairments in executive processes, including inhibitory control, cognitive flexibility, working memory, planning, emotion regulation, and other forms of hot executive functioning such as emotional decision-making and reward-related control, are consistently associated with difficulties in everyday functioning. These findings challenge diagnostic models based primarily on symptom clusters and support the view that alterations in executive functioning may reflect a shared neurocognitive vulnerability across mental disorders. Conceptualizing executive dysfunction as a transdiagnostic dimension may therefore help integrate insights from cognitive neuroscience, clinical psychiatry, and dimensional models of psychopathology.

At the same time, executive dysfunction is unlikely to be present in all individuals or to play the same role across psychiatric conditions. Its expression may vary depending on the interaction between genetic vulnerability, environmental influences, and illness trajectories. In some cases, executive impairments may also emerge as downstream consequences of chronic emotional distress, prolonged illness, medication effects, or neurodevelopmental factors. Longitudinal evidence further suggests that the relationship between executive functioning and psychopathology may be bidirectional, with emotional symptoms sometimes predicting subsequent declines in executive functioning. These observations indicate that executive dysfunction should not be interpreted as a uniform causal factor but rather as a dynamic process that may both contribute to, and result from, psychopathological processes.

**Figure 1:**
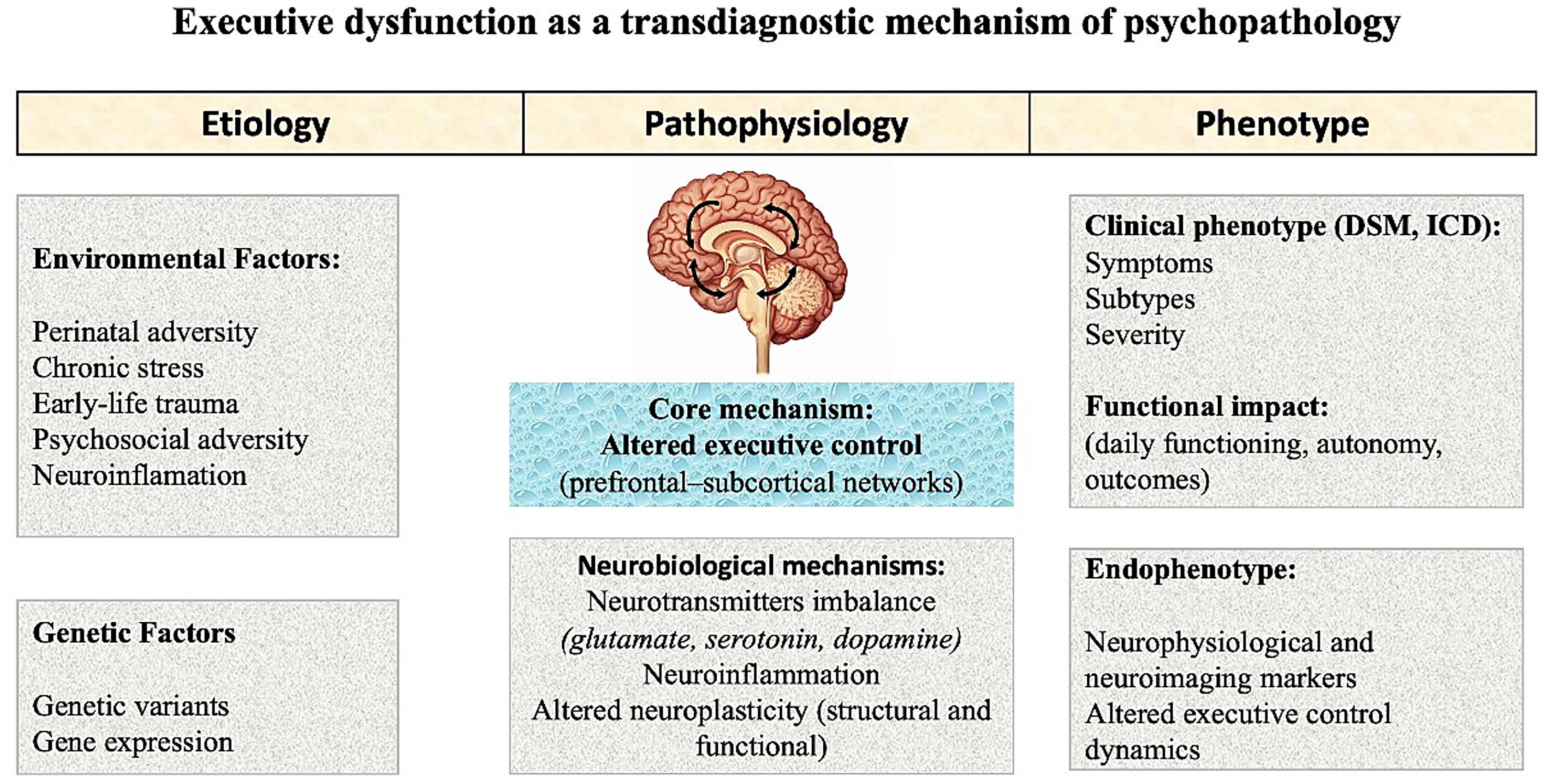
Transdiagnostic framework of executive dysfunction in psychopathology.

The framework proposed here accommodates this complexity by conceptualizing executive dysfunction as a convergent pathway through which diverse etiological influences—genetic, developmental, and environmental—translate into impairments in goal-directed behavior and real-world functioning. As illustrated in Figure 1, the model integrates the evidence reviewed in this article into a multilevel account of psychopathology. Rather than assuming that specific risk factors map directly onto diagnostic categories, the framework emphasizes convergence: genetic predisposition and environmental challenges are proposed to influence shared neurobiological pathways, particularly those involving prefrontal–subcortical control systems, which in turn shape executive functioning and clinical outcomes. From this perspective, executive functioning emerges as a key domain for assessment, theoretical investigation, and intervention in mental health research and clinical practice. Conceptualizing executive dysfunction in this way provides a unifying framework for understanding how diverse biological and environmental risk factors may converge on common cognitive mechanisms that influence functioning across diagnostic categories. Disruptions in executive functioning may act as a functional bridge between biological processes, such as neurotransmitter imbalance, neuroinflammatory activity, and altered neuroplasticity, and variability in symptoms, severity, and daily functioning. Differences in clinical presentation may therefore reflect variations in how executive control systems are disrupted or compensated for, rather than fundamentally distinct underlying mechanisms. An important implication of this model is that functional impairment cannot be understood solely in terms of symptom profiles. Limitations in executive functioning, such as difficulties sustaining goals, regulating emotions, integrating reward signals into decision-making, adapting behavior to changing demands, or initiating purposeful action, often represent key constraints on everyday functioning across psychiatric conditions. As illustrated in Figure 1, executive control systems link brain-based vulnerabilities with real-world adaptation.

Consistent with this view, executive functioning is often more strongly associated with functional outcomes than symptom severity alone. Deficits in planning, cognitive flexibility, inhibitory control, working memory, and emotion regulation are closely related to academic and occupational performance, social functioning, autonomy, and long-term prognosis across diagnostic groups and stages of illness. These findings align with dimensional frameworks such as the RDoC, which emphasize core neurocognitive systems in understanding disability and recovery.

At the neural level, executive dysfunction appears to reflect disruptions in distributed control networks rather than isolated abnormalities in single brain regions. Across disorders characterized by executive impairments, converging evidence implicates alterations in dorsolateral, ventromedial, and cingulate prefrontal regions, together with fronto-subcortical and limbic circuits. These systems support goal maintenance, self-regulation, and adaptive decision-making, and they interact with reward- and valuation-related networks that shape emotional decision-making and motivational control. From this perspective, executive dysfunction can be understood as a disturbance in the coordinated neural architecture that organizes behavior across time and context.

### 4.1 Toward new paradigms of intervention

From a clinical perspective, the framework proposed here suggests reconsidering how psychiatric interventions are conceptualized and evaluated. If executive dysfunction represents a shared mechanism underlying functional impairment across disorders, interventions targeting executive processes may have broad therapeutic relevance beyond diagnosis-specific treatments. Most current treatments are organized around diagnostic categories and focus primarily on symptom reduction. However, growing evidence, particularly from schizophrenia research, shows that symptom improvement does not consistently translate into better everyday functioning. In contrast, cognitive and executive functioning are more strongly associated with real-world outcomes such as work performance, social functioning, and independent living (Bowie and Harvey, 2006; Harvey et al., 2019). Clinically, it is common to observe individuals who meet criteria for symptomatic remission but continue to experience persistent difficulties in planning, organization, emotional regulation, and goal-directed behavior. If executive dysfunction represents a shared vulnerability across psychiatric disorders, executive processes should be considered primary therapeutic targets rather than secondary concerns. This perspective shifts the focus of intervention from disorder-specific symptoms to the cognitive systems that support adaptive functioning across contexts.

A growing body of research supports transdiagnostic, function-oriented interventions aimed at strengthening executive capacities. Cognitive remediation and executive skills training have been shown to improve functional outcomes in conditions such as schizophrenia, mood disorders, and ADHD, even when symptom changes are modest (Bowie and Harvey, 2006; Kofler et al., 2018; Snyder et al., 2015). Interventions targeting emotion regulation—including cognitive reappraisal training, mindfulness-based approaches, and acceptance-based strategies—have demonstrated effectiveness across diagnostic categories (Joormann and D’Avanzato, 2010). These approaches tend to be most effective when tailored to individuals’ executive profiles and real-world functional demands rather than guided solely by diagnostic labels. In this framework, treatment success is defined not only by symptom reduction but also by improvements in self-regulation, autonomy, and everyday functioning. This perspective aligns with evidence indicating that executive functioning predicts occupational performance, social integration, and independent living more reliably than symptom severity across psychiatric conditions (Barch and Ceaser, 2012; Millan et al., 2012).

Executive dysfunction also has important implications for treatment engagement. Many psychotherapeutic approaches implicitly assume intact executive capacities for planning, maintaining goals, monitoring behavior, and implementing learned strategies in everyday contexts. When these abilities are compromised, even evidence-based treatments may show limited effectiveness (Harvey and Strassnig, 2012). For example, difficulties in working memory, inhibitory control, or cognitive flexibility may interfere with the ability to apply therapeutic strategies, sustain behavioral change, or integrate feedback across sessions. Interventions targeting executive functioning may therefore serve a dual role: directly addressing functional impairments while also enhancing individuals’ capacity to benefit from other therapeutic approaches.

Emerging evidence further indicates that executive functioning can be modified through cognitive training, behavioral interventions, and neuromodulation targeting prefrontal networks. These approaches have shown transfer effects beyond trained tasks, leading to broader improvements in cognitive control and functional outcomes (Millan et al., 2012; Teixeira-Santos et al., 2022). These findings support a shift toward intervention models that target executive systems as core mechanisms underlying adaptive functioning. Aligning treatment strategies with the cognitive processes that support goal-directed behavior may improve long-term outcomes, reduce relapse risk, and enhance quality of life across psychiatric conditions.

### 4.2 Bridging cognition and biology: emerging genetic evidence

Over the past decade, advances in psychiatric genetics have reshaped understanding of the biological basis of mental disorders. Rather than supporting disorder-specific genetic causes, large-scale genomic studies consistently show substantial overlap in genetic risk across psychiatric conditions. Analyses including hundreds of thousands to over 1 million individuals indicate that disorders such as schizophrenia, bipolar disorder, major depression, ADHD, and anxiety disorders cluster along partially overlapping dimensions of genetic liability rather than forming distinct biological categories (Cross-Disorder Group of the Psychiatric Genomics Consortium, 2019; Selzam et al., 2018).

This pattern parallels evidence for a general psychopathology factor, or p-factor, reflecting a broad vulnerability to mental illness that cuts across diagnoses and may help explain high levels of comorbidity (Allegrini et al., 2019; Caspi et al., 2014). Many genetic variants associated with this shared risk influence biological processes involved in brain development, synaptic plasticity, and neural circuit maturation (Smoller et al., 2019; Sullivan and Geschwind, 2019). These processes are particularly relevant for prefrontal and fronto-subcortical networks that support executive control systems. Within this framework, executive dysfunction may represent a cognitive expression of shared genetic vulnerability. Rather than arising solely as a consequence of illness or treatment, difficulties in executive functioning may constitute a pathway through which genetic risk affects behavior and everyday functioning. This interpretation aligns with clinical observations that individuals with different diagnoses often show similar difficulties in planning, organization, emotional regulation, and persistence (Harvey et al., 2019; Snyder et al., 2015).

In sum, genetic and cognitive evidence supports a multilevel model in which broad genetic risk influences the development of neural circuits supporting executive control systems. In this view, executive dysfunction may serve as a key mechanism linking shared biological vulnerability to functional impairment across psychiatric disorders.

### 4.3 Implications for mental health systems

Conceptualizing executive dysfunction as a transdiagnostic process has important implications for how mental health care is organized and evaluated. This perspective highlights the potential value of incorporating executive-function-informed assessment and intervention strategies into routine mental health care. In clinical practice, difficulties in inhibition, planning, working memory, and emotion regulation are often treated as secondary issues, with the expectation that they will improve once symptoms decrease. However, many individuals continue to experience substantial functional difficulties despite symptomatic improvement. Problems with organizing daily routines, sustaining effort, regulating emotions, or maintaining goal-directed behavior often remain major sources of disability. When executive limitations are not directly addressed, functional recovery may stall. Targeting executive and self-regulatory processes therefore represents an important opportunity for improving long-term outcomes. Interventions that strengthen executive capacity may support greater functional stability, reduce relapse risk, and improve adaptation, particularly in individuals with recurrent, comorbid, or clinically complex presentations. From this perspective, executive dysfunction can be understood as a key bottleneck that limits recovery across diagnostic categories.

Focusing on executive processes also offers practical advantages for mental health systems. Patients are frequently treated sequentially across diagnostic frameworks, often receiving interventions that produce modest symptom improvements without substantially improving everyday functioning. An executive-focused perspective may help bridge these gaps by targeting shared mechanisms that influence engagement, learning, and behavioral change across conditions. Such an approach supports more mechanism-informed models of care, where assessment and intervention are guided not only by diagnostic categories but also by an individual’s capacity for planning, emotional regulation, and goal maintenance. This perspective does not replace existing diagnostic systems but complements them by embedding diagnosis within a broader neurocognitive and functional framework. Integrating executive-function-informed assessment and intervention may also support more efficient allocation of resources by prioritizing treatments that improve functional capacity. Ultimately, approaching executive dysfunction as a transdiagnostic organizing principle may contribute to more personalized, scalable, and functionally meaningful mental health care.

## 5 Conclusion

This article advances the hypothesis that executive dysfunction represents a transdiagnostic core dimension of mental disorders. Rather than viewing executive difficulties as secondary consequences of specific diagnoses or symptoms, the framework proposed here conceptualizes them as a central mechanism through which shared biological vulnerability manifests in everyday functioning. By integrating evidence from cognitive neuroscience, neuropsychology, genetics, and clinical research, the model provides a framework for understanding why diverse psychiatric conditions converge on similar patterns of functional impairment and highlights executive functioning as a potential target for transdiagnostic research and intervention.

Examining psychopathology through the lens of executive functioning shifts attention from isolated symptom clusters toward the cognitive systems that support goal-directed behavior, emotional regulation, and adaptive emotional and motivational decision-making. This perspective reflects a common clinical observation: individuals with different diagnoses frequently experience similar difficulties in domains such as autonomy, persistence, emotional control, and effective self-management. In this context, executive dysfunction may represent a key pathway linking heterogeneous clinical presentations with shared functional outcomes. If supported by future empirical research, this framework has important implications for both theory and practice. It encourages a move beyond symptom-centered models toward a neurocognitively informed, function-oriented approach to mental health. By linking genetic vulnerability, neural circuit disruption, cognitive processes, and intervention strategies within a unified framework, this perspective may support the development of more personalized, scalable, and effective approaches to assessment and treatment. Ultimately, focusing on executive functioning and its underlying control systems may provide a practical pathway toward improving the outcomes that matter most in mental health care: sustained functioning, autonomy, and quality of life.

## Data Availability

The original contributions presented in the study are included in the article/supplementary material, further inquiries can be directed to the corresponding author.

## References

[ref1] Abramovitch A. Abramowitz J. S. Mittelman A. (2013). The neuropsychology of adult obsessive-compulsive disorder: a meta-analysis. Clin. Psychol. Rev. 33, 1163–1171. doi: 10.1016/j.cpr.2013.09.004, 24128603

[ref2] Aldao A. Nolen-Hoeksema S. Schweizer S. (2010). Emotion-regulation strategies across psychopathology: a meta-analytic review. Clin. Psychol. Rev. 30, 217–237. doi: 10.1016/j.cpr.2009.11.004, 20015584

[ref3] Allegrini A. G. Selzam S. Rimfeld K. von Stumm S. Pingault J. B. Plomin R. (2019). Genomic prediction of cognitive traits in childhood and adolescence. Mol. Psychiatry 24, 819–827. doi: 10.1038/s41380-019-0394-4, 30971729 PMC6986352

[ref4] Badre D. D'Esposito M. (2009). Is the rostro-caudal axis of the frontal lobe hierarchical? Nat. Rev. Neurosci. 10, 659–669. doi: 10.1038/nrn2667, 19672274 PMC3258028

[ref5] Barch D. M. Ceaser A. (2012). Cognition in schizophrenia: core psychological and neural mechanisms. Trends Cogn. Sci. 16, 27–34. doi: 10.1016/j.tics.2011.11.015, 22169777 PMC3860986

[ref6] Barkley R. A. (1997). Behavioral inhibition, sustained attention, and executive functions: constructing a unifying theory of ADHD. Psychol. Bull. 121, 65–94. doi: 10.1037/0033-2909.121.1.65, 9000892

[ref7] Barkley R. A. (2012). Executive functions: what they are, how they work, and why they evolved. New York: Guilford Press.

[ref8] Barkley R. A. (2015). “Emotional dysregulation is a core component of ADHD,” in Attention-deficit hyperactivity disorder: a handbook for diagnosis and treatment, ed. Barkley R. A.. 4th ed (New York: The Guilford Press), 81–115.

[ref9007] Bechara A. (2005). Decision making, impulse control and loss of willpower to resist drugs: a neurocognitive perspective. Nat. Neurosci. 8, 1458–1463. doi: 10.1038/nn1584 16251988

[ref9008] Bechara A. Damasio A. R. (2005). The somatic marker hypothesis: A neural theory of economic decision. Games Econ. Behav. 52, 336–372. doi: 10.1016/j.geb.2004.06.010

[ref9] Bickel W. K. Jarmolowicz D. P. Mueller E. T. Koffarnus M. N. Gatchalian K. M. (2012). Excessive discounting of delayed reinforcers as a trans-disease process contributing to addiction and other disease-related vulnerabilities: emerging evidence. Pharmacol. Ther. 134, 287–297. doi: 10.1016/j.pharmthera.2012.02.004, 22387232 PMC3329584

[ref10] Bostan A. C. Dum R. P. Strick P. L. (2013). Cerebellar networks with the cerebral cortex and basal ganglia. Trends Cogn. Sci. 17, 241–254. doi: 10.1016/j.tics.2013.03.003, 23579055 PMC3645327

[ref11] Bowie C. R. Harvey P. D. (2006). Cognitive deficits and functional outcome in schizophrenia. Neuropsychiatr. Dis. Treat. 2, 531–536. doi: 10.2147/nedt.2006.2.4.531, 19412501 PMC2671937

[ref9005] Bowie C. R. McGurk S. R. Mausbach B. Patterson T. L. Harvey P. D. (2012). Combined cognitive remediation and functional skills training for schizophrenia: effects on cognition, functional competence, and real-world behavior. Am J Psychiatry. 169, 710–718. doi: 10.1176/appi.ajp.2012.11091337 22581070

[ref12] Brockett A. T. Pribut H. J. Vázquez D. Roesch M. R. (2018). The impact of drugs of abuse on executive function: characterizing long-term changes in neural correlates following chronic drug exposure and withdrawal in rats. Learn. Mem. (Cold Spring Harb. N.Y.) 25, 461–473. doi: 10.1101/lm.047001.117, 30115768 PMC6097763

[ref13] Caspi A. Houts R. M. Belsky D. W. Goldman-Mellor S. J. Harrington H. Israel S. . (2014). The p factor: one general psychopathology factor in the structure of psychiatric disorders? Clin. Psychol. Sci. 2, 119–137. doi: 10.1177/2167702613497473, 25360393 PMC4209412

[ref14] Coelho C. G. Leite J. Pinto R. Machado P. P. P. Carvalho S. (2025). From cortex to cardiac response: tDCS of the prefrontal cortex improves autonomic markers of emotion regulation. Brain Sci. 15:898. doi: 10.3390/brainsci15090898, 41008259 PMC12467847

[ref15] Cotrena C. Damiani Branco L. Ponsoni A. Samamé C. Milman Shansis F. Paz Fonseca R. (2020). Executive functions and memory in bipolar disorders I and II: new insights from meta-analytic results. Acta Psychiatr. Scand. 141, 110–130. doi: 10.1111/acps.13121, 31697843

[ref16] Cross-Disorder Group of the Psychiatric Genomics Consortium (2019). Genomic relationships, novel loci, and pleiotropic mechanisms across eight psychiatric disorders. Cell 179:e11, 1469–1482. doi: 10.1016/j.cell.2019.11.020, 31835028 PMC7077032

[ref17] Cuthbert B. N. (2014). The RDoC framework: facilitating transition from ICD/DSM to dimensional approaches that integrate neuroscience and psychopathology. World Psychiatry 13, 28–35. doi: 10.1002/wps.20087, 24497240 PMC3918011

[ref18] Diamond A. (2013). Executive functions. Annu. Rev. Psychol. 64, 135–168. doi: 10.1146/annurev-psych-113011-143750, 23020641 PMC4084861

[ref19] Doebel S. (2020). Rethinking executive function and its development. Perspect. Psychol. Sci. 15, 942–956. doi: 10.1177/1745691620904771, 32348707

[ref20] Duncan J. (2010). The multiple-demand (MD) system of the primate brain: mental programs for intelligent behaviour. Trends Cogn. Sci. 14, 172–179. doi: 10.1016/j.tics.2010.01.004, 20171926

[ref21] Etkin A. Egner T. Kalisch R. (2011). Emotional processing in anterior cingulate and medial prefrontal cortex. Trends Cogn. Sci. 15, 85–93. doi: 10.1016/j.tics.2010.11.004, 21167765 PMC3035157

[ref22] Everitt B. J. Robbins T. W. (2016). Drug addiction: updating actions to habits to compulsions ten years on. Annu. Rev. Psychol. 67, 23–50. doi: 10.1146/annurev-psych-122414-033457, 26253543

[ref23] Feeser M. Prehn K. Kazzer P. Mungee A. Bajbouj M. (2014). Transcranial direct current stimulation enhances cognitive control during emotion regulation. Brain Stimul. 7, 105–112. doi: 10.1016/j.brs.2013.08.006, 24095257

[ref24] Friedman N. P. Miyake A. (2017). Unity and diversity of executive functions: individual differences as a window on cognitive structure. Cortex 86, 186–204. doi: 10.1016/j.cortex.2016.04.023, 27251123 PMC5104682

[ref25] Garcia-Villamisar D. Dattilo J. Garcia-Martinez M. (2017). Executive functioning in people with personality disorders. Curr. Opin. Psychiatry 30, 36–44. doi: 10.1097/YCO.0000000000000299, 27798484

[ref26] Goldstein R. Z. Volkow N. D. (2011). Dysfunction of the prefrontal cortex in addiction: neuroimaging findings and clinical implications. Nat. Rev. Neurosci. 12, 652–669. doi: 10.1038/nrn3119, 22011681 PMC3462342

[ref27] Gonçalves Ó. F. Soares J. M. Carvalho S. Leite J. Ganho A. Fernandes-Gonçalves A. . (2015). Brain activation of the defensive and appetitive survival systems in obsessive compulsive disorder. Brain Imaging Behav. 9, 255–263. doi: 10.1007/s11682-014-9303-2, 24760279

[ref28] Grant J. E. Chamberlain S. R. (2014). Impulsive action and impulsive choice across substance and behavioral addictions: cause or consequence? Addict. Behav. 39, 1632–1639. doi: 10.1016/j.addbeh.2014.04.022, 24864028

[ref29] Grotzinger A. D. Werme J. Peyrot W. J. Frei O. de Leeuw C. Bicks L. K. . (2026). Mapping the genetic landscape across 14 psychiatric disorders. Nature 649, 406–415. doi: 10.1038/s41586-025-09820-3, 41372416 PMC12779569

[ref30] Harvey P. D. Strassnig M. (2012). Predicting the severity of everyday functional disability in people with schizophrenia: cognitive deficits, functional capacity, symptoms, and health status. World Psychiatry 11, 73–79. doi: 10.1016/j.wpsyc.2012.05.004, 22654932 PMC3363376

[ref31] Harvey P. D. Strassnig M. T. Silberstein J. (2019). Prediction of disability in schizophrenia: symptoms, cognition, and self-assessment. J. Exp. Psychopathol. 10:2043808719865693. doi: 10.1177/2043808719865693

[ref32] Hiser J. Koenigs M. (2018). The multifaceted role of the ventromedial prefrontal cortex in emotion, decision making, social cognition, and psychopathology. Biol. Psychiatry 83, 638–647. doi: 10.1016/j.biopsych.2017.10.030, 29275839 PMC5862740

[ref33] Insel T. Cuthbert B. Garvey M. Heinssen R. Pine D. S. Quinn K. . (2010). Research domain criteria (RDoC): toward a new classification framework for research on mental disorders. Am. J. Psychiatry 167, 748–751. doi: 10.1176/appi.ajp.2010.09091379, 20595427

[ref34] Joormann J. D’Avanzato C. (2010). Emotion regulation in depression: examining the role of cognitive processes: Cognition & Emotion lecture at the 2009 ISRE meeting. Cogn. Emot. 24, 913–939. doi: 10.1080/02699931003784939

[ref35] Kessler R. C. Angermeyer M. Anthony J. C. DE Graaf R. Demyttenaere K. Gasquet I. . (2007). Lifetime prevalence and age-of-onset distributions of mental disorders in the World Health Organization’s world mental health survey initiative. World Psychiatry 6, 168–176, 18188442 PMC2174588

[ref36] Kofler M. J. Sarver D. E. Harmon S. L. Moltisanti A. Aduen P. A. Soto E. F. . (2018). Working memory and organizational skills problems in ADHD. J. Child Psychol. Psychiatry 59, 57–67. doi: 10.1111/jcpp.12773, 28714075 PMC5729117

[ref37] Kofler M. J. Sarver D. E. Spiegel J. A. Day T. N. Harmon S. L. Wells E. L. (2017). Heterogeneity in ADHD: neurocognitive predictors of peer, family, and academic functioning. Child Neuropsychol. 23, 733–759. doi: 10.1080/09297049.2016.1205010, 27472007 PMC6083022

[ref38] Kräplin A. Joshanloo M. Wolff M. Krönke K. M. Goschke T. Bühringer G. . (2022). The relationship between executive functioning and addictive behavior: new insights from a longitudinal community study. Psychopharmacology 239, 3507–3524. doi: 10.1007/s00213-022-06224-3, 36190537 PMC9584881

[ref39] Leichsenring F. Steinert C. Rabung S. Ioannidis J. P. A. (2022). The efficacy of psychotherapies and pharmacotherapies for mental disorders in adults: an umbrella review and meta-analytic evaluation of recent meta-analyses. World Psychiatry 21, 133–145. doi: 10.1002/wps.20941, 35015359 PMC8751557

[ref40] Lin H. Y. Fung H. Wang Y. Ho R. C. Chen S. A. (2025). A functional magnetic resonance imaging investigation of hot and cool executive functions in reward and competition. Sensors (Basel) 25:806. doi: 10.3390/s25030806, 39943445 PMC11820429

[ref41] Martel M. M. Levinson C. A. Langer J. K. Nigg J. T. (2016). A network analysis of developmental change in ADHD symptom structure from preschool to adulthood. Clin. Psychol. Sci. 4, 988–1001. doi: 10.1177/2167702615618664, 28083448 PMC5222575

[ref42] McTeague L. M. Goodkind M. S. Etkin A. (2016). Transdiagnostic impairment of cognitive control in mental illness. J. Psychiatr. Res. 83, 37–46. doi: 10.1016/j.jpsychires.2016.08.001, 27552532 PMC5107153

[ref43] McTeague L. M. Huemer J. Carreon D. M. Jiang Y. Eickhoff S. B. Etkin A. (2017). Identification of common neural circuit disruptions in cognitive control across psychiatric disorders. Am. J. Psychiatry 174, 676–685. doi: 10.1176/appi.ajp.2017.16040400, 28320224 PMC5543416

[ref44] Millan M. J. Agid Y. Brüne M. Bullmore E. T. Carter C. S. Clayton N. S. . (2012). Cognitive dysfunction in psychiatric disorders: characteristics, causes and the quest for improved therapy. Nat. Rev. Drug Discov. 11, 141–168. doi: 10.1038/nrd3628, 22293568

[ref45] Miller E. K. Cohen J. D. (2001). An integrative theory of prefrontal cortex function. Annu. Rev. Neurosci. 24, 167–202. doi: 10.1146/annurev.neuro.24.1.167, 11283309

[ref46] Miyake A. Friedman N. P. Emerson M. J. Witzki A. H. Howerter A. Wager T. D. (2000). The unity and diversity of executive functions and their contributions to complex "frontal lobe" tasks: a latent variable analysis. Cogn. Psychol. 41, 49–100. doi: 10.1006/cogp.1999.0734, 10945922

[ref47] Niendam T. A. Laird A. R. Ray K. L. Dean Y. M. Glahn D. C. Carter C. S. (2012). Meta-analytic evidence for a superordinate cognitive control network subserving diverse executive functions. Cogn. Affect. Behav. Neurosci. 12, 241–268. doi: 10.3758/s13415-011-0083-5, 22282036 PMC3660731

[ref48] Ochsner K. N. Gross J. J. (2005). The cognitive control of emotion. Trends Cogn. Sci. 9, 242–249. doi: 10.1016/j.tics.2005.03.010, 15866151

[ref49] Rodas J. A. Leon-Rojas J. Rooney B. (2024). Mind over mood: exploring the executive function's role in downregulation. Front. Psychol. 15:1322055. doi: 10.3389/fpsyg.2024.1322055, 38333058 PMC10850342

[ref510] Rolls E. T. (2019). The orbitofrontal cortex and emotion in health and disease, including depression. Neuropsychol. 128, 14–43. doi: 10.1016/j.neuropsychologia.2017.09.021 28951164

[ref50] Romer A. L. Pizzagalli D. A. (2021). Is executive dysfunction a risk marker or consequence of psychopathology? A test of executive function as a prospective predictor and outcome of general psychopathology in the adolescent brain cognitive development study®. Dev. Cogn. Neurosci. 51:100994. doi: 10.1016/j.dcn.2021.100994, 34332330 PMC8340137

[ref51] Ruocco A. C. (2005). The neuropsychology of borderline personality disorder: a meta-analysis and review. Psychiatry Res. 137, 191–202. doi: 10.1016/j.psychres.2005.07.004, 16297985

[ref52] Sambol S. Suleyman E. Scarfo J. Ball M. (2023). A true reflection of executive functioning or a representation of task-specific variance? Re-evaluating the unity/diversity framework. Acta Psychol. 236:103934. doi: 10.1016/j.actpsy.2023.103934, 37156119

[ref53] Saxena A. Hartman C. A. Blatt S. D. Fremont W. P. Glatt S. J. Faraone S. V. . (2024). Reward functioning in general and specific psychopathology in children and adults. J. Atten. Disord. 28, 77–88. doi: 10.1177/10870547231201867, 37864336

[ref54] Seeley W. W. Menon V. Schatzberg A. F. Keller J. Glover G. H. Kenna H. . (2007). Dissociable intrinsic connectivity networks for salience processing and executive control. J. Neurosci. 27, 2349–2356. doi: 10.1523/JNEUROSCI.5587-06.2007, 17329432 PMC2680293

[ref55] Selzam S. Coleman J. R. I. Caspi A. Moffitt T. E. Plomin R. (2018). A polygenic p factor for major psychiatric disorders. Transl. Psychiatry 8:205. doi: 10.1038/s41398-018-0217-4, 30279410 PMC6168558

[ref56] Shankar S. Shyamprakash J. Ravishankar K. S. Rahamathulla M. M. I. (2025). Executive dysfunction and working memory in borderline personality disorder and their association with impulsivity and self-injurious behavior: a cross-sectional study. Indian J. Psychol. Med. 2025:4096. doi: 10.1177/02537176251364096, 40832653 PMC12357828

[ref57] Shaw P. Stringaris A. Nigg J. Leibenluft E. (2014). Emotion dysregulation in attention deficit hyperactivity disorder. Am. J. Psychiatry 171, 276–293. doi: 10.1176/appi.ajp.2013.13070966, 24480998 PMC4282137

[ref58] Smoller J. W. Andreassen O. A. Edenberg H. J. Faraone S. V. Glatt S. J. Kendler K. S. (2019). Psychiatric genetics and the structure of psychopathology. Mol. Psychiatry 24, 409–420. doi: 10.1038/s41380-017-0010-4, 29317742 PMC6684352

[ref59] Snyder H. R. (2013). Major depressive disorder is associated with broad impairments on neuropsychological measures of executive function: a meta-analysis and review. Psychol. Bull. 139, 81–132. doi: 10.1037/a0028727, 22642228 PMC3436964

[ref60] Snyder H. R. Miyake A. Hankin B. L. (2015). Advancing understanding of executive function impairments and psychopathology: bridging the gap between clinical and cognitive approaches. Front. Psychol. 6:328. doi: 10.3389/fpsyg.2015.00328, 25859234 PMC4374537

[ref61] Stuss D. T. (2011). Functions of the frontal lobes: relation to executive functions. J. Int. Neuropsychol. Soc. 17, 759–765. doi: 10.1017/S1355617711000695, 21729406

[ref62] Stuss D. T. Alexander M. P. (2007). Is there a dysexecutive syndrome? Philos. Trans. R. Soc. Lond. Ser. B Biol. Sci. 362, 901–915. doi: 10.1098/rstb.2007.2096, 17412679 PMC2430005

[ref63] Sullivan P. F. Geschwind D. H. (2019). Defining the genetic, genomic, cellular, and diagnostic architectures of psychiatric disorders. Cell 177, 162–183. doi: 10.1016/j.cell.2019.01.015, 30901538 PMC6432948

[ref64] Teigset C. M. Mohn C. Rund B. R. (2020). Perinatal complications and executive dysfunction in early-onset schizophrenia. BMC Psychiatry 20:103. doi: 10.1186/s12888-020-02517-z, 32131788 PMC7057649

[ref65] Teixeira-Santos A. C. Moreira C. S. Pereira D. R. Pinal D. Fregni F. Leite J. . (2022). Working memory training coupled with transcranial direct current stimulation in older adults: a randomized controlled experiment. Front. Aging Neurosci. 14:827188. doi: 10.3389/fnagi.2022.827188, 35493937 PMC9039392

[ref66] Tolonen T. Leppämäki S. Roine T. Alho K. Tani P. Koski A. . (2024). Working memory related functional connectivity in adult ADHD and its amenability to training: a randomized controlled trial. Neuroimage Clin. 44:103696. doi: 10.1016/j.nicl.2024.103696, 39536524 PMC11602582

[ref67] Tubío Fungueiriño M. Fernández Prieto M. Carvalho S. Leite J. Carracedo Á. Gonçalves Ó. F. (2020). Executive impairments in obsessive compulsive disorder: a systematic review with emotional and non-emotional paradigms. Psicothema 1, 24–32. doi: 10.7334/psicothema2019.187, 31954412

[ref68] Willcutt E. G. Doyle A. E. Nigg J. T. Faraone S. V. Pennington B. F. (2005). Validity of the executive function theory of attention-deficit/hyperactivity disorder: a meta-analytic review. Biol. Psychiatry 57, 1336–1346. doi: 10.1016/j.biopsych.2005.02.006, 15950006

[ref69] Zainal N. H. Newman M. G. (2023). Elevated anxious and depressed mood relates to future executive dysfunction in older adults: a longitudinal network analysis of psychopathology and cognitive functioning. Clin. Psychol. Sci. 11, 218–238. doi: 10.1177/21677026221114076, 36993876 PMC10046395

[ref70] Zelazo P. D. Carlson S. M. (2012). Hot and cool executive function in childhood and adolescence: development and plasticity. Child Dev. Perspect. 6, 354–360. doi: 10.1111/j.1750-8606.2012.00246.x

